# Effects of blue pulsed light on human physiological functions and subjective evaluation

**DOI:** 10.1186/1880-6805-31-23

**Published:** 2012-09-03

**Authors:** Tetsuo Katsuura, Yukifumi Ochiai, Toshihiro Senoo, Soomin Lee, Yoshika Takahashi, Yoshihiro Shimomura

**Affiliations:** 1 Graduate School of Engineering, Chiba University, Chiba, Japan; 2 Advanced Technology Development Center, Health and Environment Systems Group, SHARP Corporation, Osaka, Japan; 3 Present address: Center for Environment, Health and Field Sciences, Chiba University, Chiba, Japan

**Keywords:** Blue pulsed light, LED, Non-visual effect, Pupillary light response, Subjective evaluation

## Abstract

**Background:**

It has been assumed that light with a higher irradiance of pulsed blue light has a much greater influence than that of light with a lower irradiance of steady blue light, although they have the same multiplication value of irradiance and duration. We examined the non-visual physiological effects of blue pulsed light, and determined whether it is sensed visually as being blue.

**Findings:**

Seven young male volunteers participated in the study. We placed a circular screen (diameter 500 mm) in front of the participants and irradiated it using blue and/or white light-emitting diodes (LEDs), and we used halogen lamps as a standard illuminant. We applied three steady light conditions of white LED (F0), blue LED + white LED (F10), and blue LED (F100), and a blue pulsed light condition of a 100-μs pulse width with a 10% duty ratio (P10). The irradiance of all four conditions at the participant's eye level was almost the same, at around 12 μW/cm^2^. We measured their pupil diameter, recorded electroencephalogram readings and Kwansei Gakuin Sleepiness Scale score, and collected subjective evaluations. The subjective bluish score under the F100 condition was significantly higher than those under other conditions. Even under the P10 condition with a 10% duty ratio of blue pulsed light and the F10 condition, the participant did not perceive the light as bluish. Pupillary light response under the P10 pulsed light condition was significantly greater than under the F10 condition, even though the two conditions had equal blue light components.

**Conclusions:**

The pupil constricted under the blue pulsed light condition, indicating a non-visual effect of the lighting, even though the participants did not perceive the light as bluish.

## Introduction

Recently, quite a few studies in the field of physiological anthropology have focused on the non-visual effects of illumination on humans [1-5], with findings such as increased sympathetic nervous activity and improved arousal level, which are generally seen in response to high-color-temperature illumination that contains a rich short-wavelength light.

In 2002, intrinsically photosensitive retinal ganglion cells (ipRGCs), a novel type of photoreceptor cell, were found in the mammalian retina [6]. Researchers found that ipRGCs affect the suprachiasmatic nucleus and act as the primary photoreceptor for non-visual effects, such as the suppression of pineal melatonin synthesis [7,8] and pupillary constriction [9-14]. It was also found that ipRGCs respond to short-wavelength light of around 480 nm (blue light) [6,15].

Although the density of ipRGCs in the retina is much lower than that of rods or cones, resulting in a very low photon catch, each captured photon elicits a large and extraordinarily prolonged response [16]. It was reported that the contribution of ipRGCs to the pupillary light response was greater at a higher irradiance, with the role of the rods being more dominant at a lower irradiance level [9,12,14]. Because of this characteristic of ipRGCs, it was assumed that the light condition with higher irradiance of pulsed blue light had a much greater influence than that of the light condition with lower irradiance of steady blue light, although the two conditions had the same multiplication value of irradiance and duration. There have been no studies on the non-visual effect of blue pulsed light, including on the pupillary response.

 The purpose of this study was to clarify the visual and non-visual features of blue pulsed light by examining the physiological functions and subjective impressions under several lighting conditions, including pulsed and steady lighting of blue light-emitting diodes (LEDs).

## Methods

**Figure 1:**
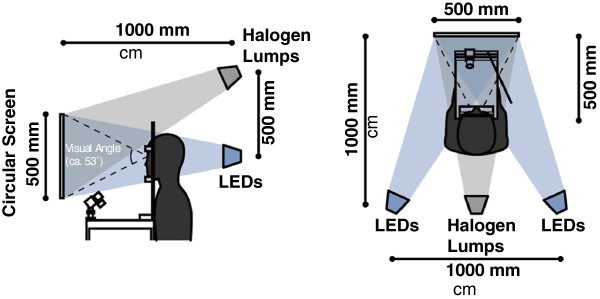
Experimental configuration.

Seven healthy young male Japanese volunteers (22 ± 0.7 years old) with dark eyes participated in the study. They were screened for normal color vision, using the Farnsworth Munsell 100 Hue Test. Each participant gave his informed consent to participate in the study. The Ethics Committee of the Graduate School of Engineering, Chiba University approved the protocol of the study. The experiment was conducted in a climatic chamber (TBR-6HA4G2C; ESPEC Corp., Osaka, Japan) in which the air temperature and relative humidity were set at 26 °C and 50%, respectively. Each participant sat on a chair with his head fixed on a positioner 500 mm from a circular screen 500 mm in diameter. We irradiated the circular screen using blue LEDs (NS6B083T-W; Nichia Corp., Anan, Japan) and white LEDs (NS6L183T-H3; Nichia Corp., Anan, Japan), and we used a halogen lamp as a standard illuminant (Figure 1). The correlated color temperature of the halogen lamp was 3,114 K, and that of the white LED was 3,034 K.

We applied four light conditions, of which three were steady and one was pulsed. The three steady light conditions were white LEDs (F0), blue and white LEDs (F10), and blue LEDs (F100). The pulsed light (P10) was blue LEDs with a 100-μs pulse width in a square waveform plus white LEDs. The time ratio of the blue LED lighting was 10% of the duty ratio, that is, the blue LEDs were turned on for 100 μs and the white LEDs were turned on for 900 μs alternately for every 1,000-μs interval.

**Figure 2:**
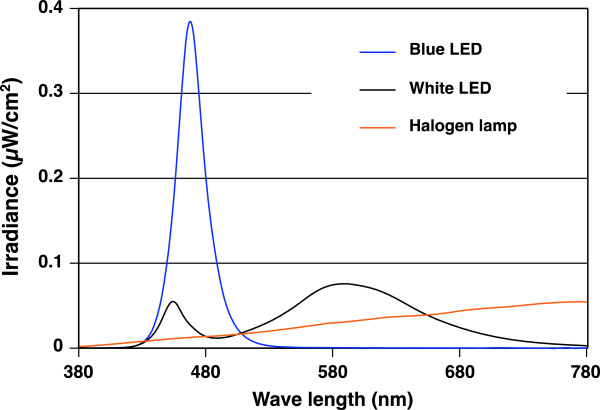
**Spectral irradiance of blue light-emitting diodes, white light-emitting diodes and a halogen lamp.** Spectral irradiance of blue light-emitting diodes and white light-emitting diodes correspond to those of the F100 condition and F0 condition.

**Figure 3:**
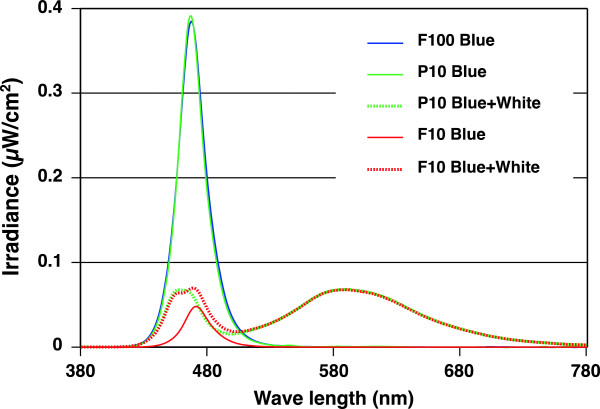
**Spectral irradiance of each light condition.** The spectral irradiance of blue LEDs in F100 condition (F100 Blue), blue LEDs in P10 condition (P10 Blue), blue and white LEDs in P10 condition (P10 Blue + White), blue LEDs in F10 condition (F10 Blue), and blue and white LEDs in F10 condition (F10 Blue + White) are shown. LED: light-emitting diode.

The spectral irradiance of blue and white LEDs and the halogen lamp (Figure 2), and that of each light condition (Figure 3) were measured at the participant's eye level by a spectroradiometer (HSR-8100; MAKI Manufacturing, Co. Ltd., Hamamatsu, Japan). The characteristics of each light condition are shown in Table 1.

**Table 1:** Characteristics of each light condition

**Condition**	**F0**	**F10**			**P10**			**F100**
**Light-emitting diode**	**White**	**Blue**	**White**	**Blue + White**	**Blue**	**White**	**Blue + White**	**Blue**
Steady/pulsed*	Steady	Steady	Steady	Steady	Pulsed			Steady
**Duty ratio (%)**	-	-	-	-	10	90	-	-
**Peak wavelength (nm)**	-	471	-	-	467	-	-	467
**Full-width at half-maximum (nm)**	-	24	-	-	23	-	-	23
**Photopic illuminance (l×)**	37.2	1.27	33.2	34.5	8.52	37.1	34.3	8.92
**Scotopic illuminance (l×)**	46.9	16.8	42.0	58.8	125	46.3	54.2	130
**Irradiance (μW/cm**^**2**^**)**	11.6	1.4	10.4	11.8	11.2	11.6	11.6	11.6
**Photon density (log photons/cm**^**2**^**/s)**	13.5	12.5	13.5	13.5	13.4	13.5	13.5	13.4

*Steady: steady light condition, Pulsed: pulsed light condition.

In the F10 condition, blue LEDs and white LEDs produced light simultaneously at the irradiance ratio of about 1:9. F10 Blue, shown in Table 1 and Figure 3, shows the properties when only blue LEDs were lit. The peak wavelength of F10 Blue was 471 nm, which was different from those of F100 and P10 Blue. However, the difference was small. P10 Blue in Figure 3 and Table 1 had values that were tenfold as great as the actual measurements of the blue LED (lighted solely at 1,000 Hz with 100-μs pulse width) in consideration of 10% of the duty ratio. The peak wavelength and full-width at half-maximum of P10 Blue were the same as those of the blue LED or F100 condition. The spectral irradiance of F10 and P10 conditions in which blue and white LEDs produced light are shown in Figure 3 as ‘F10 Blue + White’ and ‘P10 Blue + White’ respectively. The peak around 460 to 470 nm of F10 conditions was slightly shifted toward the long wavelength from that of the P10 condition due to the different properties of blue light.

The irradiance of all four conditions at the participant’s eye level was almost the same, around 12 μW/cm^2^, as shown in Table 1. The photon density, photopic illumination and scotopic illumination of each light condition are also described in Table 1.

In the experiment, the participant rested for six minutes, underwent an alpha attenuation task for six minutes and then rested for three minutes in the standard illuminant condition. He then rested for nine minutes and underwent an alpha attenuation task for six minutes under each of the four lighting conditions. We measured pupil diameter (EMR-8B, nac Image Technology Inc., Tokyo, Japan) during the last three minutes of rest under the standard illuminant condition and during the nine minutes of rest for each lighting condition, and we then calculated the pupillary responses by subtracting mean values of the pupil diameter under the standard illuminant condition from the mean values under the experimental condition. We also analyzed data from electroencephalograms (EEGs) at Fz, Cz and Pz electrode sites of the international 10–20 system, from the Kwansei Gakuin Sleepiness Scale and from the visual analog scale for subjective evaluations of sleepiness, concentration, fatigue and bluish during the experiment. One or two of the four light conditions was performed per day at the same time of day on three different days. The order of the four light conditions was counterbalanced among the participants.

One-way repeated measures analysis of variance was used to evaluate the effects of the light condition on these measurement values. When any significant effect was found, multiple comparisons of the light condition were performed by Holm's sequential Bonferroni procedure [17]. The level of statistical significance was set at 0.05.

## Results and discussion

**Figure 4:**
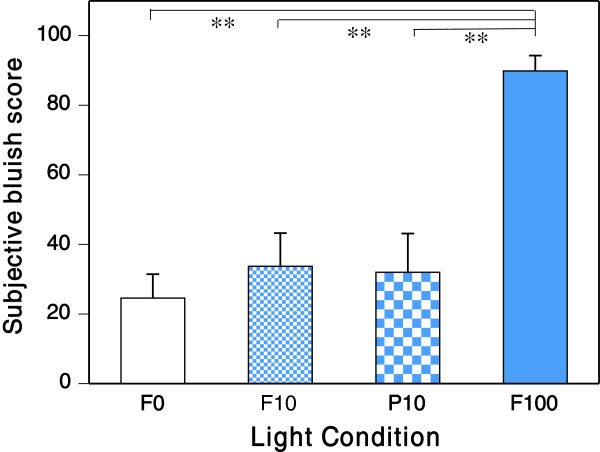
Subjective bluish score under F0, F10, P10 and F100 conditions (mean ± standard error). **P< 0.01.

We found that the subjective bluish score under the blue LED 100% condition (F100) was significantly higher (*P* < 0.01) than those under other conditions. Even under the P10 condition with a 10% duty ratio of blue pulsed light of 11.2 μW/cm^2^ and the F10 condition including a steady blue LED of 1.4 μW/cm^2^, the participant did not perceive the light as bluish (Figure 4). No main effects of light condition on Kwansei Gakuin Sleepiness Scale score or subjective sleepiness, concentration or fatigue were found.

**Figure 5:**
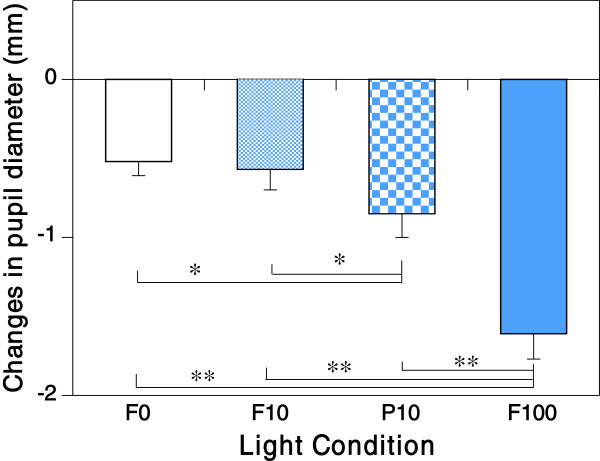
Changes in pupil diameter under F0, F10, P10 and F100 conditions (mean ± standard error). *P < 0.05; **P < 0.01.

Change in the pupil diameter under the F100 condition was significantly greater (*P* < 0.01) than under other conditions, and showed pronounced pupillary response. Moreover, we found that the pupillary response under the P10 pulsed light condition was significantly larger ( *P* < 0.05) than under the F10 and F0 conditions (Figure 5).

Changes in the EEG alpha wave band ratio at Pz under the F100 condition tended to be lower (*P* < 0.1) than under the F10 condition. There were no significant effects of light condition on the alpha attenuation coefficient.

In our study, the most important finding was that pupillary response under the P10 pulsed light condition was significantly greater than under the F10 condition, although the subjective bluish score under the P10 condition was as low as those under the F0 and the F10 conditions.

The melanopsin-stimulating irradiance and photon density at the participant's retinal level of each light condition were also estimated [18] based on the spectral absorption of the crystalline lens [19] and a template [20] indicating the spectral absorption characteristics of the photopigment with peak wavelength of 484 nm [6] (Table 2).

**Table 2:** Melanopsin-stimulating irradiance at retinal level of each light condition

**Condition**	**F0**	**F10**			**P10**			**F100**
**Light-emitting diode**	**White**	**Blue**	**White**	**Blue + White**	**Blue**	**White**	**Blue + White**	**Blue**
**Melanopsin-stimulating irradiance (μW/cm**^**2**^**)**	2.29	1.17	2.06	3.22	8.84	2.25	2.91	9.18
**Melanopsin-stimulating photon density (photons/cm**^**2**^**/s)**	5.65 × 10^12^	2.89 × 10^12^	5.08 × 10^12^	7.97 × 10^12^	21.8 × 10^12^	5.57 × 10^12^	7.19 × 10^12^	22.7 × 10^12^

The melanopsin-stimulating irradiance and photon density at retinal level of the F10 (3.22 μW/cm^2^ and 7.97 × 10^12^ photons/cm^2^/s) and the P10 conditions (2.91 μW/cm^2^ and 7.19 × 10^12^ photons/cm^2^/s) calculated from the multiplication values of irradiance and duration were almost the same. Despite this, the pupillary response under the P10 condition was significantly greater than under the F10 condition. This might have been due to the short (100 μs) but higher melanopsin-stimulating irradiance features of the blue pulsed light of the P10 condition (8.84 μW/cm^2^ and 21.8 × 10^12^ photons/cm^2^/s) as compared with the blue component of the F10 condition (1.17 μW/cm^2^ and 2.89 × 10^12^ photons/cm^2^/s) as shown in Table 2. It has been suggested that pupillary light response is controlled by rods under lower irradiance light exposure and by ipRGCs under higher irradiance light exposure [9,14]. [ Panda *et al*. 9] reported that ipRGC contributed to the pupillary response in mice at an irradiance level greater than about 13 log photons/cm^2^/s of 470 nm light at their eye level. [Takahashi *et al*. 14] also estimated that the irradiance level at which the role of rods in pupillary constriction was replaced by ipRGCs was around 4.54 μW/cm^2^ or 46.0 scotopic lx at the participant's eye level. In addition, it was reported from experiments on rats that the threshold retinal irradiance for depolarization of ipRGCs was about 12.7 log photons/cm^2^/s of 500 nm light at eye level [6]. In our study, irradiance at the eye level of P10 Blue (13.4 log photons/cm^2^/s or 125 scotopic lx) was greater than those values of irradiance. Because of this, we propose that the blue pulsed light of strong irradiance included in the P10 condition induced significantly greater pupillary response.

## Conclusion

In the present study, we found for the first time that the pupillary response under a 100-μs pulsed blue light condition was significantly greater than under a steady blue light condition with equal blue light components, even though the participants did not perceive the light as bluish under these conditions.

## Abbreviations

EEG, Electroencephalogram; ipRGC, Intrinsically photosensitive retinal ganglion cell; LED, Light-emitting diode.

## Competing interests

The authors declare that they have no competing interests.

## Authors’ contributions

TK conceived and designed the study, and wrote the manuscript. YO performed the experiments, analyzed the data, and wrote the first draft. TS devised the light-control devices. TK, TS, SL, YT and YS were responsible for coordination of the study and overseeing data collection and analysis. All authors have read and approved the final manuscript.
